# Network Completion for Static Gene Expression Data

**DOI:** 10.1155/2014/382452

**Published:** 2014-03-26

**Authors:** Natsu Nakajima, Tatsuya Akutsu

**Affiliations:** Bioinformatics Center, Institute for Chemical Research, Kyoto University, Gokasho, Uji, Kyoto 611-0011, Japan

## Abstract

We tackle the problem of completing and inferring genetic networks under stationary conditions from static data, where network completion is to make the minimum amount of modifications to an initial network so that the completed network is most consistent with the expression data in which addition of edges and deletion of edges are basic modification operations. For this problem, we present a new method for network completion using dynamic programming and least-squares fitting. This method can find an optimal solution in polynomial time if the maximum indegree of the network is bounded by a constant. We evaluate the effectiveness of our method through computational experiments using synthetic data. Furthermore, we demonstrate that our proposed method can distinguish the differences between two types of genetic networks under stationary conditions from lung cancer and normal gene expression data.

## 1. Introduction

Estimation of genetic interactions from gene expression microarray data is an interesting and important issue in bioinformatics. There are two kinds of gene expression data: time series data and nontime series data. To estimate the dynamics of gene regulatory networks such as cell cycle and life cycle processes, various mathematical models and methods have been proposed using time series data. Since the number of observed time points in time series data is usually small, these methods suffer from low accuracies. On the other hand, a large number of nontime series data are available, for example, samples from normal people and patients of various types of diseases. Although these data are not necessarily static, we may regard these data as static data because these are averaged over a large amount of cells in rather steady states.

For inference of genetic networks, various reverse engineering methods have been proposed, which include methods based on Boolean networks [1, 2], Bayesian networks [3, 4], differential equations [5–7], and graphical Gaussian models [8–10]. Boolean networks can only be applied to inference of genetic networks from time series data because the Boolean network is intrinsically a dynamic model. Although Bayesian networks have widely been applied to analysis of static data, they can only output acyclic networks. Many methods have also been proposed using various kinds of differential equation models. However, in many cases, parameter estimation needs a huge amount of computation time. Overall, most methods suffer from inaccuracy and/or computational inefficiency and thus there is not yet an established or standard method for inference of genetic networks using only gene expression data. Therefore, it is reasonable to try to develop another approach for analysis of gene regulatory networks.

In recent years, there have been several studies and attempts for network completion, not necessarily for biological networks but also for social networks and web graphs. Different from network inference, we assume in network completion that a certain type of a prototype network is given, which can be obtained by using existing knowledge. Kim and Leskovec [11] addressed the network completion problem in which an incomplete network including unobserved nodes and edges is given and then the unobserved parts should be inferred. They proposed KronEM, which combined the Expectation Maximization with the Kronecker graphs model to estimate the missing part of the network. Guimerà and Sales-Pardo [12] presented a mathematical and computational method which can identify both missing and spurious interactions in complex networks by using the stochastic block models to capture the structural features in the network. This method was also applied to a protein interaction network of yeast. Hanneke and Xing [13] defined the network completion as a problem of inferring the rest part of the network, given an observed incomplete network sample and proposed a sampling method to derive confidence intervals from sample networks. As a related work, Saito et al. [14] developed a method to measure the consistency of an inferred network with the measured gene expression data.

Independently, Akutsu et al. [15] proposed another model of network completion in which the objective is to make the minimum amount of modifications to a given network so that the resulting network is most consistent with the observed data. Based on this concept, Nakajima et al. [16] developed a practical method, DPLSQ, for completion of genetic networks from time series data, in which addition and deletion of edges are the basic modification operations and the numbers of added and deleted edges are specified. In addition, if we begin with a network with an empty set of edges, it can be applied to network inference. DPLSQ is based on a combination of least-squares fitting and dynamic programming, where least-squares fitting is used for estimation of parameters in differential equations and dynamic programming is used for minimizing the sum of least-squares errors under the restriction of the number of added and deleted edges. Different from other heuristic or stochastic approaches, DPLSQ is guaranteed to output an optimal solution (in the sense of the minimum least-squares error) in polynomial time if the maximum indegree of nodes is bounded by a constant. Nakajima and Akutsu [17] proposed a method to complete and infer the time varying networks by extending DPLSQ so that additions and deletions of edges can be performed at several time points. However, since DPLSQ is based on a dynamic model, it cannot be applied to inference or completion of genetic networks from static data.

In this study, we propose a novel method, DPLSQ-SS (DPLSQ for Static Samples), for completing and inferring a network using static gene expression data, based on DPLSQ. The purpose of this study is twofold: first, to complete and infer gene networks from static expression profile, instead of time series data and, secondly, to investigate the relationship between different kinds of inferred networks under different conditions (e.g., comparison of normal and cancer networks obtained from samples of normal and cancer cells). Static data typically consist of expression levels of genes, which were measured at single time point but for a large number of samples. As discussed in the beginning part of this section, these types of data can be regarded as the gene expression measurements in a stationary phase. Many of static microarray data are publicly available, in particular for cancer microarray data with a relatively large size of tumor and normal samples. Therefore, it may be possible to estimate and investigate differences between cancer and normal networks. The basic strategy of DPLSQ-SS is the same as that of DPLSQ: least-squares fitting is used for parameter estimation and dynamic programming is used for minimizing the sum of least-squares errors when adding and deleting edges. In order to cope with static data, we modified the error function to be minimized. Although the idea is simple, it brings wider applicability because a large number of static gene expression data are available. We demonstrate the effectiveness of DPLSQ-SS through computational experiments using synthetic data and gene expression data for lung cancer and normal samples. We also perform computational comparison of DPLSQ-SS as an inference method with some of state-of-the-art tools using synthetic data.

## 2. Method

**Figure 1:**
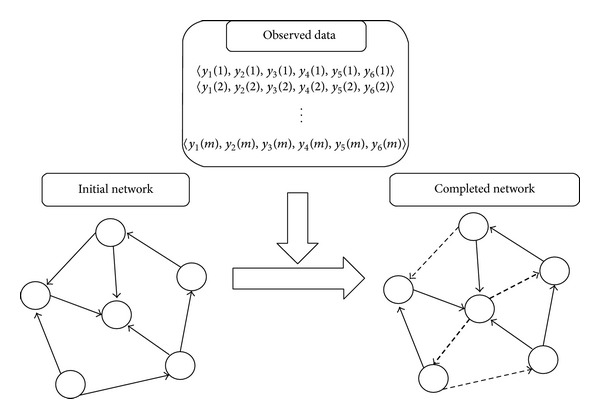
Network completion by addition and deletion of edges from *m* samples. The bold dashed and the thin dashed edges represent added and deleted edges, respectively.

The purpose of network completion in this study is to modify a given network by making the minimum number of modifications so that the resulting network is most consistent with the observed data. Here we assume additions and deletions of edges as modification operations (see Figure 1). In the following, graph *G*(*V*, *E*) denotes a given network where *V* and *E* are the sets of nodes and directed edges, including loops, respectively. In this graph *G*, each node corresponds to a gene and each edge represents a direct regulation between two genes. We let *n* denote the number of genes and let *V* = {*v*_1_,…, *v*_*n*_}. For each node *v*_*i*_, *e* ^−^(*v*_*i*_) and deg⁡^−^(*v*_*i*_), respectively, denote the set of incoming edges to *v*_*i*_ and the number of incoming edges to *v*_*i*_ as defined below:

(1) $$\begin{array}{c} e^{-}\left(v_{i}\right)=\left\{v_{j}\;|\;\left(v_{j},v_{i}\right)\in E\right\},\\ \deg^{-}\left(v_{i}\right)=\left|e^{-}\left(v_{i}\right)\right|. \end{array}$$

We employ least-squares fitting for the parameter estimation and dynamic programming for identifying structure of the network. In the following we explain the algorithm of the proposed method.

### 2.1. Model of Nonlinear Equation and Estimation of Parameters

**Figure 2:**
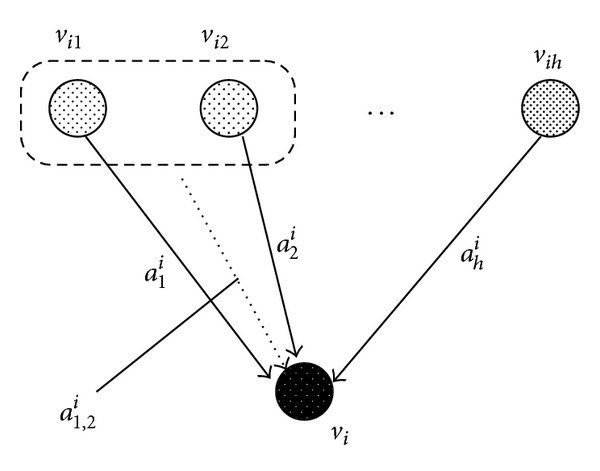
Static model of genetic network. The expression level of *v*_*i*_ is determined from those of input nodes. *a*_1,2_^*i*^ is a coefficient corresponding to cooperative regulation by genes *v*_*i*_1__ and *v*_*i*_2__ to gene *v*_*i*_.

Since we consider static data in this paper, we adopt a mathematical model based on nonlinear equations, instead of differential equations in [16]. We assume that the static state of each node *v*_*i*_ is determined by the following equation:

(2) $$\begin{array}{c} x_{i}=a_{0}^{i}+\sum_{j=1}^{h}a_{j}^{i}x_{i_{j}}+\sum_{j\leq k}a_{j,k}^{i}x_{i_{j}}x_{i_{k}}+b^{i}\omega , \end{array}$$

where *v*_*i*_1__,…, *v*_*i*_*h*__ are incoming nodes to *v*_*i*_, *x*_*i*_ corresponds to the expression value of the *i*th gene, and *ω* denotes a random noise. The second and third terms of the right-hand side of the equation represent linear and nonlinear effects to node *v*_*i*_, respectively (see Figure 2), where positive *a*_*j*_^*i*^ or *a*_*j*,*k*_^*i*^ corresponds to an activation effect and negative *a*_*j*_^*i*^ or *a*_*j*,*k*_^*i*^ corresponds to an inhibition effect.

We assume that static expression data 〈*y*_1_(*s*), *y*_2_(*s*),…, *y*_*n*_(*s*)〉, *s* = 1,…, *m*, are given, where *m* is the number of samples and *y*_*i*_(*s*) denotes the expression value of node *v*_*i*_ in the *s*th sample. The parameters (i.e., *a*_0_^*i*^, *a*_*j*_^*i*^, *a*_*j*,*k*_^*i*^) can be estimated by minimizing the following objective function using a standard least-squares fitting method:

(3) Si1,i2,…,ihi =∑s=1m|yi(s)−(a0i+∑j=1hajiyij(s)+∑j≤kaj,kiyij(s)yik(s))|2.

### 2.2. Completion by Addition of Edges

Once the objective function is determined, the completion procedure is the same as that for DPLSQ [16]. In order to make this paper self-contained, we also present the completion procedure here. For the simplicity, we begin with network completion by adding *k* edges in total so that the sum of least-squares errors is minimized.

We let *σ*_*k*_*j*_,*j*_^+^ denote the minimum least-squares error when adding *k*_*j*_ edges to the *j*th node and they are defined as

(4) $$\begin{array}{c} \sigma_{k_{j},j}^{+}=\underset{j_{1},j_{2},\ldots ,j_{k_{j}}}{\min}S_{j_{1},j_{2},\ldots ,j_{k_{j}}}^{j}, \end{array}$$

where each *v*_*j*_*l*__ must be selected from *V* − *v*_*j*_ − *e* ^−^(*v*_*j*_). In order to avoid combinatorial explosion, we constrain the maximum *k*_*j*_ to be a small constant *K* and let *σ*_*k*_*j*_,*j*_^+^ = +*∞* for *k*_*j*_ > *K* or *k*_*j*_ + deg⁡^−^(*v*_*j*_) ≥ *n*.

Here, we define *D* ^+^[*k*, *i*] by

(5) $$\begin{array}{c} D^{+}\left[k,i\right]=\underset{k_{1}+k_{2}+\cdots +k_{i}=k}{\min}\sum_{j=1}^{i}\sigma_{k_{j},j}^{+}. \end{array}$$

The entries of *D* ^+^[*k*, *i*] can be computed by the dynamic programming algorithm as follows:

(6) $$\begin{array}{c} D^{+}\left[k,1\right]=\sigma_{k,1}^{+},\\ D^{+}\left[k,j+1\right]=\underset{k^{\prime }+k^{\prime \prime }=k}{\min}\left\{D^{+}\left[k^{\prime },j\right]+\sigma_{k^{\prime \prime },j+1}^{+}\right\}. \end{array}$$

It is to be noted that *D* ^+^[*k*, *n*] is determined uniquely regardless of the ordering of nodes in the network. The correctness of this dynamic programming algorithm can be seen by

(7) min⁡k1+k2+⋯+kn=k∑j=1nσkj,j+ =min⁡k′+k′′=k{min⁡k1+k2+⋯+kn−1=k′∑j=1n−1σkj,j++σk′′,n+} =min⁡k′+k′′=kD+[k′,n−1]+σk′′,n+.

### 2.3. Completion by Addition and Deletion of Edges

The above dynamic programming procedure can be modified for addition and deletion of edges.

We let *σ*_*k*_*j*_,*h*_*j*_,*j*_ denote the minimum least-squares error when adding *k*_*j*_ edges to *e* ^−^(*v*_*j*_) and deleting *h*_*j*_ edges from *e* ^−^(*v*_*j*_), where added and deleted edges must be disjoint. As described in Section 2.2, we also constrain the maximum *k*_*j*_ and *h*_*j*_ to be small constants *K* and *H*, respectively. We let *σ*_*k*_*j*_,*h*_*j*_,*j*_ = +*∞* if *k*_*j*_ > *K*, *h*_*j*_ > *H*, *k*_*j*_ − *h*_*j*_ + deg⁡^−^(*v*_*j*_) ≥ *n*, or *k*_*j*_ − *h*_*j*_ + deg⁡^−^(*v*_*j*_) < 0 holds. Then, the problem is stated as

(8) $$\begin{array}{c} \underset{\begin{array}{c} k_{1}+k_{2}+\cdots +k_{n}=k\\ h_{1}+h_{2}+\cdots +h_{n}=h \end{array}}{\min}\sum_{\;j=1}^{n}\sigma_{k_{j},h_{j},j}. \end{array}$$

Here, we define *D*[*k*, *h*, *i*] by

(9) $$\begin{array}{c} D\left[k,h,i\right]=\underset{\begin{array}{c} k_{1}+k_{2}+\cdots +k_{i}=k\\ h_{1}+h_{2}+\cdots +h_{i}=h \end{array}}{\min}\sum_{\;j=1}^{i}\sigma_{k_{j},h_{j},j}. \end{array}$$

Then, the network completion problem by addition and deletion of edges can be solved by using the dynamic programming algorithm as follows:

(10) $$\begin{array}{c} D\left[k,h,1\right]=\sigma_{k,h,1},\\ D\left[k,h,j+1\right]=\underset{\begin{array}{c} k^{\prime }+k^{\prime \prime }=k\\ h^{\prime }+h^{\prime \prime }=h \end{array}}{\min}\left\{D\left[k^{\prime },h^{\prime },j\right]+\sigma_{k^{\prime \prime },h^{\prime \prime },j+1}\right\}. \end{array}$$

We will also discuss the computational complexity of DPLSQ-SS. Since completion by addition of edges is a special case, we only analyze completion by addition and deletion of edges.

It is known that least-squares fitting for a linear system can be done in *O*(*mp* ^2^ + *p* ^3^) time where *m* is the number of samples and *p* is the number of parameters. In our proposed method, we assume that the maximum indegree in a given network and the number of parameters are bounded by constants. In this case, the time complexity per least-squares fitting can be estimated as *O*(*m*).

Next we analyze the time complexity required for *σ*_*k*_*j*_,*h*_*j*_,*j*_ and *D*[*k*, *h*, *i*]. The time complexity required for computation of *σ*_*k*_*j*_,*h*_*j*_,*j*_ is *O*(*mn* ^*K*+1^) [16], where the time complexity of computing the minimum least-squares for *j*th node depends on the upper bounds for the number of adding and deleting edges per node, *K* and *H*. In addition, the time complexity for *D*[*k*, *h*, *i*]s is *O*(*n* ^3^) [16], considering that the size of table *D*[*k*, *h*, *i*] is *O*(*n* ^3^). Therefore, total time complexity for DPLSQ-SS is

(11) $$\begin{array}{c} O\left({mn}^{K+1}+n^{3}\right). \end{array}$$

This analysis suggests that DPLSQ-SS can be applicable to large-scale networks if *K* ≤ 2 and *n* is not too large.

If the maximum indegree of the initial network is not bounded by a constant, the time complexity per least-squares fitting increases to *O*(*mn* ^4^ + *n* ^6^) and the number of combinations to be examined per node increases to *O*(*n* ^*H*+*K*^), as discussed in [16]. In this case, the total time complexity would be *O*(*n* ^*H*+*K*+1^ · (*mn* ^4^ + *n* ^6^)), which suggests that network completion should not start with dense networks but with sparse networks.

## 3. Results

To evaluate the effectiveness of DPLSQ-SS, we performed two types of computational experiments using both synthetic data and real expression data. All experiments were performed on a PC with Intel Core 2 Quad CPU (3.0 GHz). We employed the liblsq library (http://www2.nict.go.jp/aeri/sts/stmg/K5/VSSP/install_lsq.html) for a least-squares fitting method.

### 3.1. Inference Using Synthetic Data

In order to assess the potential effectiveness of DPLSQ-SS, we begin with network inference using two kinds of synthetic data. Recall that network completion beginning with a null network corresponds to network inference.

We employed here nonlinear equations as gene regulation rules between genes. Since it is difficult to generate static data by numerical simulations, we made manually nonlinear equations with obvious solutions as the synthetic network topology and regarded each solution as static data for one sample. For example, if we make *n* equations with *n* variables, it is assumed that there exist *n* genes in the synthetic network. We give an example of nonlinear equations with 3 variables below:

(12) x1=x12−2,x2=x22−6,x3=x1x2−1,

where we assume that *x*_*i*_  (*i* = 1,…, 3) corresponds to the expression value of *i*th gene. Therefore, an example network consists of 3 genes and 4 edges, including self-loops. If we solve this set of equations, we can find four solutions as below:

(13) $$\begin{array}{c} \left(2,3,5\right),\;\;\left(2,-2,-5\right),\;\;\left(-1,3,-4\right),\;\;\left(-1,-2,1\right). \end{array}$$

Then, we can employ these solutions as synthetic data. Since the use of synthetic static data consisting only of a few solutions easily resulted in numerical calculation error, we generated additional 400 data sets for each of static solutions by adding random numbers uniformly distributed between −0.5 and 0.5.

Under the above model, we examined DPLSQ-SS for network inference, using synthetic data which is generated as described above and letting *E* = *∅* in the initial network. It should be noted that we let upper bounds for the number of adding and deleting edges per node to be *K* = 2 and *H* = 0, respectively. Furthermore, in order to examine the CPU time changes with respect to the size of the network, we made synthetic networks with 10 and 20 nodes by making the nonlinear equation with corresponding number of variables.

Since the number of added edges was always equal to the number of edges in the original network, we evaluated the performance of DPLSQ-SS by means of the averaged accuracy, which was defined as the number of correctly inferred edges to the number of edges in the original network (i.e., the number of added edges) and the averaged computational time over 5 modified networks.

We also compared DPLSQ-SS with two well-known existing tools for inference of genetic networks, ARACNE [18, 19] and GeneNet [9, 10]. ARACNE is based on mutual information between genes and GeneNet is based on graphical Gaussian models and partial correlations. Since both tools output only correlation values for genes, we selected the top *M* from them and regarded {*v*_*i*_, *v*_*j*_} as a correct edge if either (*v*_*i*_, *v*_*j*_) or (*v*_*j*_, *v*_*i*_) was included in the edge set of the original network. We employed datasets which were generated by the same way for DPLSQ-SS and default parameter settings for both tools. We evaluated the results by the ratio of correctly inferred edges and averaged CPU time (see Table 1). The CPU time used by ARACNE is user time + sys time and that used by GeneNet is time difference between the start time and end time.

The results on DPLSQ-SS and comparative methods using synthetic data show that the accuracies by DPLSQ-SS are higher than those by ARACNE and GeneNet. Although ARACNE cannot handle networks with self-loops but GeneNet can, both methods showed almost the same performance in the case of *n* = 10. On the whole, three methods have something in common, which perform with low accuracy as the size of the network grows. As for the CPU time, ARACNE was faster than DPLSQ-SS and GeneNet in case of *n* = 10. In addition, the CPU time by DPLSQ-SS increases rapidly as the size of the network grows, in contrast to those by the comparative methods. Since DPLSQ-SS works in polynomial time, if we obtain sufficient computer resource, DPLSQ-SS can handle large-scale networks. Since accuracy is the most important criterion and DPLSQ-SS is more accurate than existing methods, our proposed method might be a useful tool for network inference.

### 3.2. Inference Using DREAM4 Data

In this subsection, we try to evaluate the effectiveness of DPLSQ-SS and perform a comparison with other methods in order to perform an unbiased evaluation since the results in Section 3.1 are based on the mathematical model adopted by DPLSQ-SS. We used synthetic datasets generated by GeneNetWeaver (GNW) [20], which provide benchmarks and performance testing for network inference methods in the DREAM (Dialogue on Reverse Engineering Assessment and Methods) challenge (http://www.the-dream-project.org/challenges). One aim of the DREAM project is to provide benchmark data on real and simulated expression data for network inference. This challenge includes several editions, where GNW has been developed to generate genetic network motifs and simulated expression data. In this evaluation, we used the DREAM4 challenge which is divided into three subchallenges called InSilico_Size10, InSilico_Size100, and InSilico_Size100_Multifactorial, consisting of five networks.

We validated the performance using InSilico_Size10 subchallenge consisting of gold standard 10 gene networks and simulated expression data generated under different conditions (wild-type, knockouts, knockdowns, multifactorial perturbations, and time series). Since only one set of wild-type data, which corresponds to static data, is provided for each network and it is not enough for inference, we generated 500 static data sets by randomly perturbing each data as in Section 3.1. The result is shown in Table 2, where the accuracy was evaluated as in Section 3.1. It is seen from the table that the performance of any method is not good. It is reasonable because inference was preformed based on one set of expression data (i.e., *m* = 1) although perturbed data were also used. Although ARACNE was better than DPLSQ-SS in four cases, DPLSQ-SS was better than ARACNE in one case. DPLSQ-SS was better than GeneNet in four cases and was comparative to GeneNet in one case. This result suggests that although DPLSQ-SS is not necessarily the best for simulated data in DREAM4, it has reasonable performance when a very few samples are given.

### 3.3. Completion Using Synthetic Data

We also examined network completion using synthetic data. In this experiment, we adopted the nonlinear equations described in Section 3.1. In order to examine network completion, we also applied DPLSQ-SS to synthetic networks, which are generated by randomly adding *k* edges and deleting *h* edges from an original network.

We assess the DPLSQ-SS performance in terms of the accuracy of modified edges and the computational time for network completion. The accuracy is defined as follows:

(14) $$\begin{array}{c} \frac{h+k+\left|E_{\text{org}}\cap E_{\text{cmp}}\right|-\left|E_{\text{org}}\right|}{h+k}, \end{array}$$

where *E*_org_ and *E*_cmp_ are the set of edges in the original network and the completed network, respectively. This value takes 1 if all added and deleted edges are correct and takes 0 if all added and deleted edges are incorrect. For each (*k*, *h*), we took the averaged accuracy and CPU time for completing the network over 5 modifications for 10 and 20 gene networks, where we used the default values of *K* = *H* = 2. To avoid the numerical calculation error, we also generated additional 400 data sets for each of static solutions by adding random numbers uniformly distributed between −0.5 and 0.5.

The results are shown in Table 3. It is observed that DPLSQ-SS has quite high accuracy regardless of the number of *k* and *h* except for *k* = *h* = 5. It is also seen that the CPU time increases rapidly when applied to networks with 20 genes. In comparison with the CPU time for network inference by DPLSQ-SS, there seems to be a significant difference even if *n* equals 10. In this study, we used the default values of *K* = 2 and *H* = 2 for network completion, which were *K* = 2 and *H* = 0 for network inference. Moreover, the number of modified edges for network inference is much larger than that for network completion. However, the latter procedure requires more CPU time than the former procedure. This result suggests that the time complexity of DPLSQ-SS depends not so much on the number of modified edges, *k* and *h*, but depends much on the number of *K* and *H* as indicated in Section 2.3.

### 3.4. Inference Using Real Data

**Figure 3:**
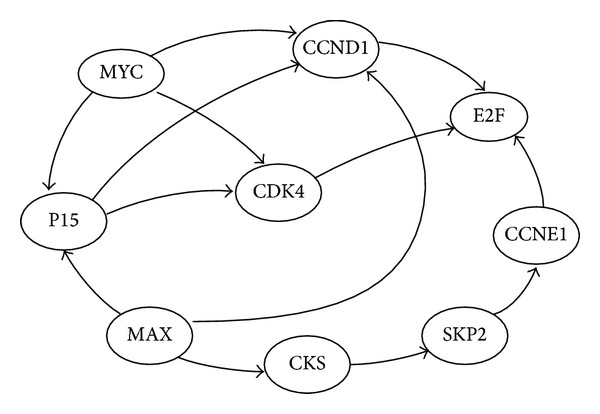
A part of small cell lung cancer network, containing RB/E2F pathway.

We also examined DPLSQ-SS for inference of gene networks from static data under multiple conditions. The aim of this experiment is to identify different static gene networks under different conditions and investigate the differences of these network topologies. We focus on the genetic network related to lung cancer and employed a partial network which contains RB/E2F pathway in human small cell lung cancer from the KEGG database [21] shown in Figure 3. RB/E2F pathway is one of two main tumor suppressor pathways and the retinoblastoma gene (RB) plays a key role in cancer [22]. RB is known to control the activity of E2F transcription factor which regulates the cell-cycle progression and E2F is under the control of both CDK4 and CCND1 (CyclinD1). It is also known that the activity of E2F plays an important role in the tumor cell proliferation and that absence of E2F leads to cancer formation. In this way, it is obvious that the gene abnormality of RB/E2F pathway is linked to cancer and there are precise differences between the cancer gene network and the normal gene network. Therefore, inferring the cancer and normal gene networks is quite meaningful. In this study, we demonstrate that DPLSQ-SS can distinguish the difference between cancer and normal networks from static expression data. Based on the KEGG database and Entrez Gene ID, we selected 9 genes shown in Table 4. We referred to RefGene (http://refgene.com/) for gene symbols and annotations and employed the resulting network as the original network.

**Figure 4:**
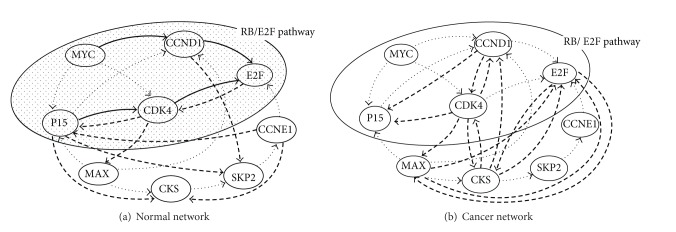
Results on inference with gene expression data for cancer and normal samples. We could verify the activation of RB/E2F pathway in the normal network and the inactivation of it in the cancer network. The bold dashed arrows denote the incorrectly added edges. The bold solid arrows denote the correctly added edges (i.e., added edges that are included in the original network). Since directions of edges are ignored in comparison with ARACNE and GeneNet, bold dashed arrows connecting to P15 are regarded as correct in the evaluation of accuracy in Table 5.

As for the static expression data, we employed lung cancer microarray data obtained by Beer et al. [23]. They clustered hierarchically the gene expression profiles from lung adenocarcinoma tumor tissues and those from normal lung tissues. This data contains 86 tumor samples and 10 normal samples and is publicly available from the study of Choi and Kendziorski [24]. In order to investigate the relationship between cancer and normal gene network topologies, we performed network inference using these two types of data, where *K* = 2, *H* = 0, and *k* = 13 were used. In order to avoid the numerical calculation error, we also generated additional 5 data sets for each expression value by adding random numbers uniformly distributed between −0.5 and 0.5. The results are shown in Figure 4.

We also compared DPLSQ-SS with ARACNE and GeneNet using these real data. The result is shown in Table 5, where the accuracy (i.e., the ratio of the number of correctly inferred edges to the number of added edges) was calculated as in Section 3.1 and the networks obtained from the KEGG database were regarded as the correct networks. Although DPLSQ-SS was worse than ARACNE for the cancer network, it was better for the normal network. For both networks, DPLSQ-SS was better than GeneNet. This result suggests that the accuracy of DPLSQ-SS for real data is reasonable compared with existing methods.

Although the accuracy of DPLSQ-SS is not high for real data, there are significant differences between cancer and normal networks. The inferred normal network indicated the existence of RB/E2F pathway involved in the regulation of E2F activity. It is observed that the tumor suppressor gene P15 regulated CDK4 activity and E2F was under the regulation of both CDK4 and CCND1. On the other hand, in the inferred tumor network, we found no significant correlations between genes in RB/E2F pathway. Instead, we discovered the regulation of CCND1 and deregulation of E2F activity. It has been reported that overexpression of CDK4/6 and CCND1 and deregulated E2F could contribute to cancer progression [22, 25]. Therefore, inference of two types of networks could produce the reasonable outcome that matches biological knowledge as mentioned above and could capture the features of each network. Although there is no common edge between the inferred cancer network and the original network, it is reasonable because cancer networks may be very different from normal networks. This result suggests that our proposed method can infer different static networks under the different conditions and can identify the feature of cancer and normal networks.

## 4. Conclusion

In this study, we addressed the problem of completing and inferring gene networks under the stationary conditions from static gene expression data. In our approach, we defined network completion as making the minimum amount of modifications to an initial network so that the inferred network is most consistent with the gene expression data. The aim of this study is (1) to complete genetic networks using static data and (2) to investigate the differences between two types of gene networks under different conditions. In order to achieve our goal, we proposed a novel method called DPLSQ-SS for network completion and network inference based on dynamic programming and least-squares fitting. This method works in polynomial time if the maximum indegree is bounded by a constant. We demonstrated the effectiveness of DPLSQ-SS through computational experiments using synthetic data and real data. In particular, we tried to infer the normal and lung cancer networks from static gene microarray data. As the results using synthetic data, DPLSQ-SS showed relatively good performance in comparison to other existing methods. As the results using microarray data from normal and lung cancer samples, it is seen that this method allows us to distinguish the differences between gene networks under different conditions.

There is some room for extending DPLSQ-SS. For example, we employed here simple nonlinear equations as gene regulation rules, but it can be replaced by more complex types of nonlinear equations. Although DPLSQ-SS works in polynomial time, the degree of polynomial is not low, which prevents the method from being applied to completion of large networks. However, DPLSQ-SS can be highly parallelizable: *σ*_*k*_*j*_,*h*_*j*_,*j*_ can be computed independently for different *σ*_*k*_*j*_,*h*_*j*_,*j*_s. Therefore, parallel implementation of DPLSQ-SS is also important future work. Although we have focused on completion and inference of gene regulatory networks, completion and inference of large-scale protein-protein or ChIP-chip/seq interaction networks are also important. Since the proposed method is only applicable to gene regulatory networks, extension and application of DPLSQ-SS for these networks should be studied in the future work.

## Figures and Tables

**Table 1:** Results on inference using synthetic data.

		Method		
		DPLSQ-SS	ARACNE	GeneNet
*n* = 10	Accuracy	0.779	0.578	0.571
	CPU time (sec.)	1.784	1.113	4.020
*n* = 20	Accuracy	0.722	0.554	0.390
	CPU time (sec.)	14.482	4.795	4.040

**Table 2:** Results on inference using DREAM4 data, where the accuracy is shown for each case.

	Method		
	DPLSQ-SS	ARACNE	GeneNet
Insilico_size_10_1	0.2666	0.2000	0.0666
Insilico_size_10_2	0.1875	0.2500	0.1250
Insilico_size_10_3	0.1333	0.2000	0.0666
Insilico_size_10_4	0.1538	0.3076	0.0769
Insilico_size_10_5	0.0833	0.1666	0.0833

**Table 3:** Results on completion with synthetic data on DPLSQ-SS.

	Number of added edges and number of deleted edges	Accuracy	CPU time (sec.)
*n* = 10	*k* = 1, *h* = 1	1.000	0.720
	*k* = 2, *h* = 2	1.000	5.810
	*k* = 3, *h* = 3	1.000	4.610
	*k* = 4, *h* = 4	1.000	5.000
	*k* = 5, *h* = 5	0.700	4.410

*n* = 20	*k* = 1, *h* = 1	1.000	2.760
	*k* = 2, *h* = 2	1.000	51.870
	*k* = 3, *h* = 3	0.833	46.220
	*k* = 4, *h* = 4	1.000	53.880
	*k* = 5, *h* = 5	0.700	48.910

**Table 4:** List of gene symbols and annotations in human small cell lung cancer network.

Number	Gene symbol	Gene annotation
1	MYC	v-myc myelocytomatosis viral oncogene homolog (avian)
2	P15	Cyclin-dependent kinase inhibitor 2B
3	CDK4/6	Cyclin-dependent kinase 4
4	CCND1	Cyclin D1
5	MAX	MYC associated factor X
6	CKS1	CDC28 protein kinase regulatory subunit 1B
7	SKP2	S-phase kinase-associated protein 2 (p45)
8	CCNE1	Cyclin E1
9	E2F3	E2F transcription factor 3

**Table 5:** Results on inference of real networks, where the accuracy is shown for each case.

	Method		
	DPLSQ-SS	ARACNE	GeneNet
Normal network	0.3846	0.3076	0.0769
Cancer network	0.1538	0.3846	0.0769
